# Editorial: Aromatic Amino Acid Metabolism

**DOI:** 10.3389/fmolb.2019.00022

**Published:** 2019-04-10

**Authors:** Qian Han, Robert S. Phillips, Jianyong Li

**Affiliations:** ^1^Key Laboratory of Tropical Biological Resources of Ministry of Education, College of Life Sciences and Pharmacy, Hainan University, Haikou, China; ^2^Department of Chemistry, University of Georgia, Athens, GA, United States; ^3^Department of Biochemistry, Virginia Tech, Blacksburg, VA, United States

**Keywords:** aromatic amino acids, metabolism, tryptophan, serotoinin, melatonin, auxin, kynurenine, dopamine

Aromatic amino acids, like other proteinogenic amino acids, are the building blocks of proteins and include phenylalanine, tryptophan, and tyrosine. All plants and micro-organisms synthesize their own aromatic amino acids to make proteins (Braus, 1991; Tzin and Galili, 2010). However, animals have lost these costly metabolic pathways for aromatic amino acids synthesis and must instead obtain the amino acids through their diet. Herbicides take advantage of this by inhibiting enzymes involved in aromatic amino acid synthesis, thereby making them toxic to plants but not to animals (Healy-Fried et al., 2007).

In animals and humans, aromatic amino acids serve as precursors for the synthesis of many biologically/neurologically active compounds that are essential for maintaining normal biological functions. Tyrosine is the initial precursor for the biosynthesis of dopa, dopamine, octopamine, norepinephrine, and epinephrine, etc., that are fundamental by functioning as neurotransmitters or hormones for animals and humans (Vavricka et al., 2010). In addition, tyrosine is the precursor for melanin synthesis in most organisms including humans and animals, and is particularly important in insects for protection (Whitten and Coates, 2017). Tryptophan is the initial precursor for the biosynthesis of tryptamine, serotonin, auxin, kynurenines, and melatonin (Hardeland and Poeggeler, 2003; Mukherjee and Maitra, 2015). Kynurenic acid, a kynurenine, produced along the tryptophan-kynurenine pathway, is an antagonist at excitatory amino acid receptors and plays a role in protecting neurons from overstimulation by excitatory neurotransmitters (Han et al., 2008). Many enzymes involved in aromatic amino acids metabolism have been drug targets for diseases including neurodegenerative diseases, schizophrenia, and cancers (Stone and Darlington, 2013; Selvan et al., 2016).

In addition, since animals or humans that do not possess the enzymatic machinery for the *de novo* synthesis of aromatic amino acids must obtain these primary metabolites from their diet, the metabolism of aromatic amino acid by both the host animal and the resident microflora are important for the health of humans and all animals. Among the array of metabolites at the interface between these microorganisms and the host is the essential aromatic amino acid tryptophan (Agus et al., 2018).

We are delighted by the updated information on aromatic amino acid metabolism covered in the articles of our research topic. Overall, the articles that were received for this topic: “Aromatic Amino Acid Metabolism,” including a collection of original research and review articles, provided updated information regarding the aromatic amino acid metabolism, and addressed their synthesis and catabolism in plants and microbes, metabolic enzymes in animals and humans, and structure and function relationships of enzymes involved in the metabolism.

A review by Parthasarathy et al. included in this topic, describes the aromatic amino acid biosynthetic pathways in plants and microbes, catabolism in plants, degradation via the monoamine and kynurenine pathways in animals, and catabolism via the 3-aryllactate and kynurenine pathways in animal-associated microbes. L-Tyrosine is an aromatic amino acid synthesized *de novo* in plants and microbes via two alternative routes mediated by a TyrA family enzyme, prephenate, or arogenate dehydrogenase, typically found in microbes and plants, respectively. In the research article by Schenck et al. it was revealed that bacterial homologs, closely-related to plant TyrAs, also have an acidic residue at position 222 and arogenate dehydrogenase activity as the plant enzyme does, which indicates that the conserved molecular mechanism operated during the evolution of arogenate-specific TyrAa in both plants and microbes. Tryptophan is another aromatic amino acid, which can be oxidized by tryptophan 2,3-dioxygenase and indoleamine 2,3 dioxygenase in the initial step in tryptophan catabolism in animals and humans. Although these two enzymes catalyze the same reaction, the assembly of the catalytically active, ternary enzyme-substrate-ligand complexes is not yet fully resolved. Nienhaus and Nienhaus summarized present knowledge of ternary complex formation in tryptophan 2,3-dioxygenase and indoleamine 2,3 dioxygenase and related these findings to structural peculiarities of their active sites. Aromatic amino acids can also be oxidized by phenylalanine, tyrosine, or tryptophan hydroxylase, and then decarboxylated by aromatic amino acid decarboxylases to form aromatic monoamines. The N-acylation of the aromatic monoamines by arylalkylamine N-acyltransferases is mostly associated with the acetylation of serotonin to form N-acetylserotonin, a precursor in the formation of melatonin (Hardeland and Poeggeler, 2003; Mukherjee and Maitra, 2015). Insects express more arylalkylamine N-acyltransferases in order to regulate aromatic amino acid metabolism (Hiragaki et al., 2015). For example, 13 putative arylalkylamine N-acyltransferases have been identified in *Aedes aegypti* (Han et al., 2012) and 8 putative arylalkylamine N-acyltransferases have been identified in *Drosophila melanogaster* (Amherd et al., 2000; Dempsey et al., 2014). O'Flynn et al. highlighted the current metabolomic knowledge of the N-acylated aromatic amino acids and N-acylated derivatives of the aromatic amino acids, the current mechanistic understanding of the arylalkylamine N-acyltransferases, and explored the possibility that arylalkylamine N-acyltransferase serve as insect “rhymezymes” regulating photoperiodism and other rhythmic processes in insects.

Aromatic amino acid metabolism also involves some pyridoxal 5′-phosphate dependent enzymes, including decarboxylases, aminotransferases, and aromatic phenylacetaldehyde synthase. In the final review article of this special issue, Liang et al. provided updated knowledge of pyridoxal 5′-phosphate dependent enzymes and summarized the structural factors that contribute to the reaction mechanisms, particularly active site residues critical for dictating the reaction specificity.

## Author Contributions

All authors listed have made a substantial, direct and intellectual contribution to the work, and approved it for publication.

### Conflict of Interest Statement

The authors declare that the research was conducted in the absence of any commercial or financial relationships that could be construed as a potential conflict of interest.
